# Effects of a Newly Developed Therapeutic Deep Heating Device Using High Frequency in Patients with Shoulder Pain and Disability: A Pilot Study

**DOI:** 10.1155/2019/8215371

**Published:** 2019-05-02

**Authors:** Gi-Wook Kim, Yu Hui Won, Sung-Hee Park, Jeong-Hwan Seo, Dae-hyun Kim, Hyung Nam Lee, Myoung-Hwan Ko

**Affiliations:** ^1^Department of Physical Medicine & Rehabilitation, Chonbuk National University Medical School, Jeonju 54907, Republic of Korea; ^2^Research Institute of Clinical Medicine of Chonbuk National University-Biomedical Research Institute of Chonbuk National University Hospital, Jeonju 54907, Republic of Korea; ^3^Translational Research & Clinical Trial Center for Medical Device, Chonbuk National University Hospital, Jeonju 54907, Republic of Korea; ^4^Department of Physical Therapy, Graduate School, Daejeon University, Daejeon 34520, Republic of Korea

## Abstract

**Objectives:**

The newly developed therapeutic deep heating device can generate deep heat in focal tissue using high-frequency wave stimulation. The objective of this study was to evaluate and compare the effectiveness of this deep heating device (HIPER-500®) with ultrasound in alleviating pain and improving function in patients with shoulder disability.

**Methods:**

This noninferiority trial was designed to compare the treatment effect of HIPER-500® to that of SonoStim® (ultrasound) on shoulder pain and disability. Thirty-eight patients with shoulder problems were assigned to either the HIPER-500® or SonoStim® group, and each participated in 10 min therapy sessions, five days a week for two weeks (for a total of ten sessions). Shoulder pain and disability were evaluated using the Visual Analogue Scale (VAS), the University of California at Los Angeles score (UCLA score), the Shoulder Pain and Disability Index (SPADI), and the Constant score in both groups before, immediately after, and four weeks after treatment. Statistical analysis was performed to compare the effects of treatment within and between the groups.

**Results:**

A total of 34 patients completed the study. The 18 patients in the HIPER-500® group and 16 patients in the SonoStim® group all showed significant improvements in shoulder pain and function when comparing pretreatment values with the results immediately after treatment and four weeks later. The results before and after treatment did not show a statistically significant difference between the two groups.

**Conclusions:**

The newly developed HIPER-500® for high-frequency deep heat therapy showed similar effects to those of SonoStim® for relieving pain and improving physical performance in the patients of this study. HIPER-500® may be a useful modality for treating shoulder pain and improving physical activity in patients with shoulder disease.

## 1. Introduction

The incidence of shoulder pain has increased significantly in recent years, which is attributed to an increase in both athletic activities and the elderly population worldwide. Although the prevalence of shoulder pain varies among reports, its lifetime prevalence has been reported to be around 50–65% globally. In Korea, the 1-year prevalence rate for shoulder pain is 4.3% of the total population and is increasing every year. Shoulder pain can be a major factor in decreasing the ability to perform daily activities by causing upper-extremity dysfunction [1–3].

There are a variety of diseases that cause shoulder pain, and various treatments for shoulder pain exist. The physical modalities to treat shoulder pain include hyperthermia, ultraviolet (UV) therapy, electrotherapy (transcutaneous electrical nerve stimulation (TENS), iontophoresis, and interferential current), and alternative therapies (vibration, light and laser therapy, and extracorporeal shock wave therapy) [4, 5]. Among the physical modalities mentioned above, it is known that deep heat treatment using ultrasonic waves and superficial heat therapy, such as a hot pack, are both effective in treating shoulder pain [6–10]. Hyperthermia as a treatment modality is aimed at a local increase in tissue temperature in a specific part of the body. Such increases in tissue temperature enhance the blood flow because of vasodilation and increased soft tissue extensibility, thus promoting tissue healing and repair by supplying proteins, nutrients, and oxygen to the damaged area [4].

Electromagnetic fields have been used medically in radiofrequency treatment devices, mostly employing microwaves around 2450 MHz. Recently, however, most of the radiofrequency energy used in the medical field ranges from 30 kHz to 30 MHz (i.e., 13.56 MHz in oncothermia, which uses a radiofrequency modulated current; 27.12 MHz in short-wave diathermy) [4, 11, 12]. In a recent study, hyperthermia induced by a wave frequency of 448 kHz was used for treatment of pain and cosmetic purposes. High-frequency waves generate heat by producing friction between tissue cells and blood. The heat generated by transcutaneous high-frequency stimulation can increase blood flow and can be expected to treat musculoskeletal pain [6]. The underlying key mechanisms are capacitive electric transfer (CET) and resistive electric transfer (RET) techniques. The CET technique works in tissue containing a high content of electrolytes such as muscle and soft tissue, and the RET technique works in tissues with higher resistance such as bones, tendons, and joints [13]. The HIPER-500® uses the CRET technique, which combines the advantages of CET and RET and applies the principle of generating heat in vivo by passing high-frequency energy between the active and inactive ceramic transducers, while increasing the frequency range (4.4 MHz) to control the depth of penetration [14–17]. In previous rat experiments using HIPER-500®, high-frequency waves increased the temperature in the muscle layer without inducing cellular or histological damage. Furthermore, high-frequency wave stimulation resulted in reduced swelling and inflammation of the injured muscle in a muscle contusion model in rats [18, 19].

The purpose of this study was to evaluate the effectiveness of the newly developed HIPER-500®, a therapeutic deep heating device using high-frequency stimulation, in reducing shoulder pain and improving shoulder function and stability, and compare the results with those of ultrasound therapy.

## 2. Materials and Methods

### 2.1. Subjects

This study was conducted after obtaining approval from the Institutional Review Board (IRB) of Chonbuk National University Hospital (IRB number: CUH 2016-08-007-002). In this prospective randomized controlled trial, all subjects were given a detailed explanation of the study, its purpose, and any ethical issues, and prior consent was obtained. The study was performed in accordance with the Declaration of Helsinki.

The patients in this study were between the age of 19 and 65 years and had had at least one month of shoulder pain. They were all able to accurately express the degree and location of their pain. The cause of shoulder pain needed to be medically diagnosed, and the patients fully understood the purpose and procedure of the study and participated in the clinical study voluntarily. Patients who were suspected to have a neurological disease or sensory deterioration during physical examination, had undergone shoulder surgery within the last six months, had received a steroid injection in the shoulder joint within the last month, or were taking oral corticosteroids or nonsteroidal anti-inflammatory drugs were excluded from the study. In addition, pregnant and lactating women, women with positive urine tests for pregnancy, women planning pregnancy during the study period, all patients with prior cardiac disease or pacemaker dependence, metal inserts, untreated severe medical conditions, and those who were unable to accurately express the pain site and intensity due to the disease or cognitive decline were excluded from this study.

The patients who remained after application of the exclusion criteria were randomly assigned a number (1 : 1 ratio) by a computer program and allocated to either the SonoStim® or the HIPER-500® group. Patients did not know which deep heat treatment would be used for their shoulder pain.

### 2.2. Interventions: Deep Heat Therapy

The lesions of the study patients were confirmed by a board-certified physiatrist using an ultrasound imaging system (Zonare Medical, Co., Ltd., South Korea) at the first visit before starting treatment. The patients were randomly treated for either ultrasound or high-frequency therapy. Ultrasound was also used to determine the exact site of treatment. The duration of treatment using either HIPER-500® or SonoStim® was 10 minutes per session and five sessions per week for two weeks, for a total of ten treatments (Table 1).

#### 2.2.1. High-Frequency Therapy (HIPER-500®)

**Figure 1:**
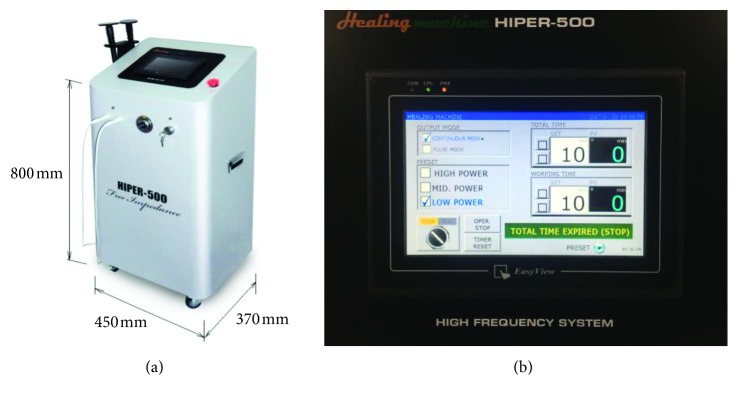
HIPER-500®, a deep heat generator (JS-ON, Co., Ltd., Seoul, South Korea). Two ceramic transducers are connected to the main device (a). By touching the screen, the user can turn the power on and off and adjust treatment intensity and time (b). Three different intensities (high, mid, and low) can be chosen (left side of the screen).

**Figure 2:**
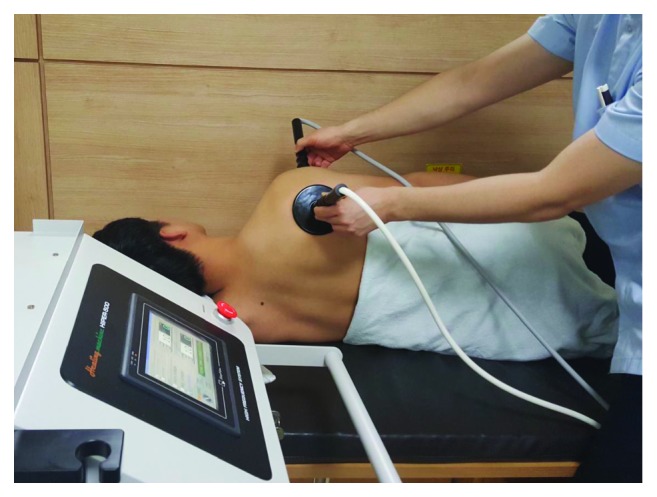
HIPER-500® treatment: the therapist moves the small ceramic transducers over the painful area while holding the large insulated plate fixed to the body.

HIPER-500® (JS-ON, Co., Ltd., Seoul, South Korea) is a high-frequency stimulation device with a size of 450 mm (width) × 370 mm (depth) × 800 mm (height). The main unit is connected with two ceramic transducers and has a monitor to indicate the status of the device and access the operation buttons (Figure 1). The HIPER-500® operates with two types of ceramic transducers, large and small, operating at a fixed frequency of 4.4 MHz with a variable power output (27, 35, and 45 W/cm^2^). Both ceramic transducers in HIPER-500 are insulated with the polyamide material of 0.5 mm thickness and mounted on a 6 mm thick aluminum plate. The large rectangular ceramic (200 mm × 150 mm) transducer is held in a fixed position on the patient's body, and the small circular ceramic (50 mm in diameter) transducer is used to move around the injury location. When both ceramic transducers are positioned near the injury, thermal energy can be effectively transferred to the affected area in both directions (Figure 2). Treatment was initially started with a moderate power output (35 W/cm^2^) and then adapted depending on how much heat the patient felt. The optimal heat intensity was defined as the highest output that the patients could tolerate. If the patients felt the heat became unbearable, the therapist reduced either the yield or the contact area between the small ceramic transducer and the skin to optimize treatment intensity. Active and continuous communication with the patients is critical to reduce side effects such as light burns and maximize the therapeutic effect with this treatment.

#### 2.2.2. Ultrasound Therapy (SonoStim®)

**Figure 3:**
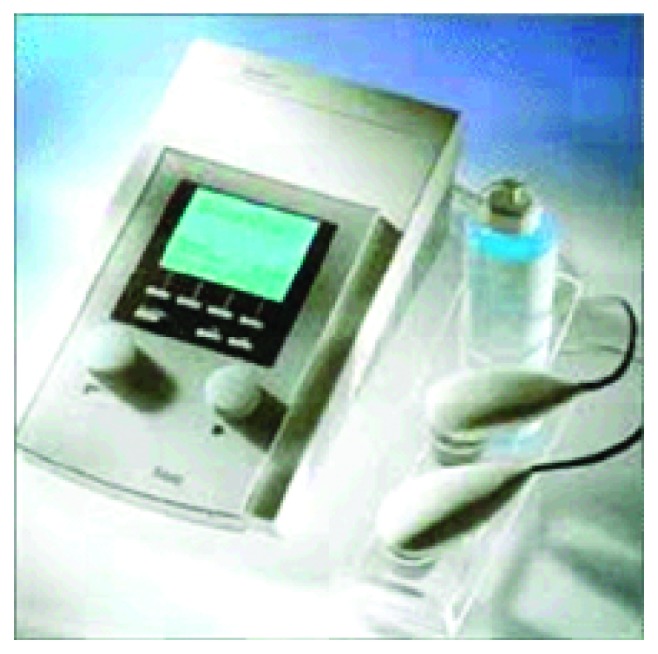
SonoStim®, a deep heat ultrasound device (Zimmer MedizinSysteme GmbH, Neu-Ulm, Germany).

Ultrasound therapy was performed using SonoStim® (Zimmer MedizinSysteme, GmbH, Neu-Ulm, Germany) (Figure 3), and the ultrasonic probe was applied to the injured location of the patient in a similar manner as in the procedure for the HIPER-500®. The frequency was set to 0.8 MHz and the power output to 2 W/cm^2^. Subsequently, the probe was moved slowly in a circular motion for optimal energy transfer to the lesion. If the patient persistently felt discomfort, this was documented as a side effect in the report.

### 2.3. Outcome Measures

**Figure 4:**
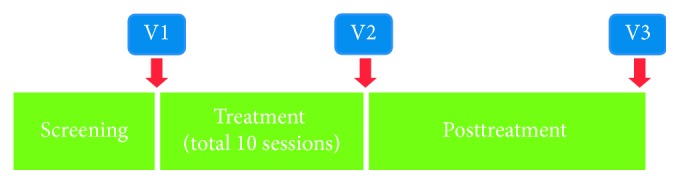
Experimental design of prospective randomized controlled trial assessing the effectiveness of deep heat therapy (*n*=34 patients). V1, visit 1, pretreatment, within 28 days after screening the patients; V2, visit 2, posttreatment period within 2 days after completing all treatment sessions; V3, visit 3, posttreatment period in 28 ± 2 days after completing all treatment sessions.

Patients were assessed at a total of four visits: screening, visit 1 (28 days after the pretreatment screening), visit 2 (posttreatment period 1, within two days after treatment), and visit 3 (posttreatment period 2, 28 ± 2 days after treatment) (Figure 4).

The Visual Analogue Scale (VAS) was used as the primary outcome measure. VAS is a subjective pain scale and the most widely used pain index. It is an evaluation tool that expresses 0 as no pain at all and 10 as the most severe pain [20]. VAS scores were differentiated into VAS-P1, pain at the current moment; VAS-P2, pain with the shoulder movement; and VAS-P3, pain in the shoulder in the resting position.

As secondary tools, the University of California at Los Angeles (UCLA) shoulder function score [17], the Shoulder Pain and Disability Index (SPADI), and the Constant score (shoulder joint treatment score) were evaluated.

The UCLA score is divided into five subscales: pain, function, active forward flexion, strength of forward flexion, and patient satisfaction with the condition of the shoulder. The maximum total score is 35 points, and the higher the score, the better the medical condition (pain and function) of the shoulder [21].

SPADI is a tool to measure shoulder pain and disability simultaneously. It consists of 13 questions: five of which are pain-related (at its worst, when lying on the side with pain, when lifting the arm to reach for objects on a high shelf, when touching the back of the neck, and when pushing something with the involved arm) and the other eight are related to physical disabilities (when washing the face or the back, when putting on underclothes or a jacket, when putting on a shirt with buttons in the front, when putting on trousers, when placing something on a high shelf, when carrying a heavy object, and when taking something out of the back pocket). The higher the score of the SPADI, the greater the degree of pain and disability [22].

Finally, the Constant score consists of 100 points and combines measures of the degree of pain and the ability to perform daily activities (maximum 35 points) with the range of motion and strength of the shoulder joint (maximum 65 points). A score of 0 indicates that the patient suffers from the most severe pain possible and cannot perform daily activities, while a score of 100 indicates a condition where the patient does not feel pain and is able to perform all daily activities [23, 24].

### 2.4. Statistical Analysis

Statistical analysis was performed using SSPS Statistics for Windows, version 18 (SSPS Inc., Chicago, IL, USA). The pretreatment (V1) and posttreatment (V2, V3) values were compared within each group, and the changes before and after treatment were compared between groups. The significance of the effect of time shown in pretreatment (V1) and posttreatment (V2, V3) in each group was analyzed using the repeated measures analysis of variance (RM ANOVA) if normality criteria of data were satisfied and the Friedman test if the assumption of normality was not satisfied. For the comparison between the two groups, the independent *t*-test was used when the assumption of normality was satisfied, and the Mann–Whitney *U*-test was used when the assumption was not satisfied. *p* < 0.05 was considered statistically significant.

## 3. Results

### 3.1. Subjects

A total of 38 patients were recruited, and 19 each were randomly assigned to either the HIPER-500® group or the SonoStim® group. In the HIPER-500® group, one participant was lost to follow-up. In the SonoStim® group, three patients did not complete the study (two follow-up losses, one adverse effect). This resulted in 18 and 16 patients completing treatment and evaluation in the HIPER-500® and SonoStim® group, respectively.

The mean age of the subjects in the HIPER-500® and the SonoStim® group was 47.83 ± 12.28 and 46.75 ± 11.50 years, respectively (*p*=0.793), and the gender ratios (male : female) were 8 : 10 and 4 : 12 in the HIPER-500® group and the SonoStim® group, respectively (*p*=0.236). Thus, age and the gender ratio were homogeneous in the two groups. The VAS-P1 was 4.17 ± 2.38 in the HIPER-500® group and 4.38 ± 0.96 in the SonoStim® group (*p*=0.736), VAS-P2 was 6.17 ± 1.86 in the HIPER-500® group and 5.13 ± 1.26 in the SonoStim® (*p*=0.068), and VAS-P3 was 2.89 ± 2.59 in the HIPER-500® group and 3.44 ± 1.59 in the SonoStim® group (*p*=0.469), showing no difference in pain between groups. The UCLA score was 24.28 ± 3.92 in the HIPER-500® group and 26.81 ± 3.54 in the SonoStim® group (*p*=0.058), SPADI-total was 41.88 ± 22.09 in the HIPER-500® group and 36.53 ± 14.84 in the SonoStim® group (*p*=0.419), and the Constant score-total was 70.04 ± 16.23 in the HIPER-500® group and 72.09 ± 10.88 in the SonoStim® group (*p*=0.673), showing no statistical significance between two groups in all evaluations (*p* > 0.05) (Table 2).

### 3.2. Outcome Measures within Both Groups

In the HIPER-500® group, VAS-P1 was measured as 4.17 ± 2.38 before treatment (V1), 2.78 ± 2.13 immediately after treatment (V2), and 2.44 ± 2.04 at 28 days after treatment (V3) (*p*=0.012), VAS-P2 was measured as 6.17 ± 1.86 before treatment (V1), 4.28 ± 2.27 immediately after treatment (V2), and 3.44 ± 2.18 at 28 days after treatment (V3) (*p*=0.001), and VAS-P3 was measured as 2.89 ± 2.59 before treatment (V1), 1.83 ± 2.09 immediately after treatment (V2), and 1.61 ± 1.72 at 28 days after treatment (V3) (*p*=0.001). In both groups, all VAS outcome measures showed a significant difference and improvement over time.

**Figure 5:**
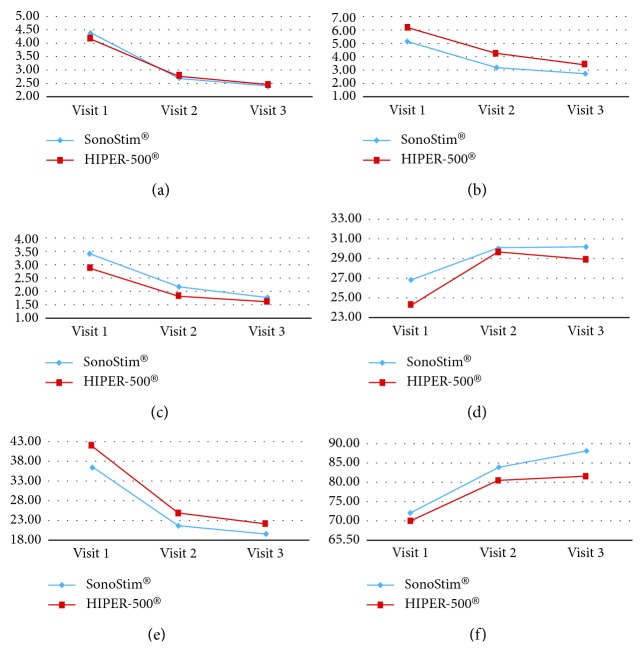
Changes in outcome measures before and after treatment in both deep heat treatment groups (*n*=34). VAS, visual analogue scale; VAS-P1, VAS pain at the current moment (a); VAS-P2, VAS pain with shoulder movement (b); VAS-P3, VAS pain in the resting position of the shoulder (c); UCLA score, University of California at Los Angeles score (d); SPADI, Shoulder Pain and Disability Index (e); Constant score-total (f).

The UCLA score was measured as a secondary outcome at 24.28 ± 3.92 before treatment (V1), 29.67 ± 3.31 immediately after treatment (V2), and 28.89 ± 3.23 at 28 days after treatment (V3) (*p* ≤ 0.001), SPADI-total was measured as 41.88 ± 22.09 before treatment (V1), 24.91 ± 17.14 immediately after treatment (V2), and 22.39 ± 15.52 at 28 days after treatment (V3) (*p*=0.005), and Constant score-total was measured as 70.04 ± 16.23 before treatment (V1), 80.36 ± 11.60 immediately after treatment (V2), and 81.41 ± 14.50 at 28 days after treatment (V3) (*p* ≤ 0.001). In both groups, all outcome measures showed a significant difference and improvement over time (Table 3, Figure 5).

In the SonoStim® group, among the evaluations measured before the start of treatment (V1), VAS-P1 as a primary outcome was 4.38 ± 0.96, VAS-P2 was 5.13 ± 1.26, and VAS-P3 was 3.44 ± 1.59, and the UCLA score as a secondary outcome was 26.81 ± 3.54, SPADI-total was 36.53 ± 14.84, and the Constant score-total was 72.09 ± 10.88. In the SonoStim® group, among the evaluations measured immediately after treatment (V2), VAS-P1 as a primary outcome was 2.69 ± 1.40, VAS-P2 was 3.19 ± 1.72, and VAS-P3 was 2.19 ± 1.33, showing statistically significant improvement in all indices, and UCLA score as a secondary outcome was 30.06 ± 3.19, SPADI-total was 22.03 ± 12.65, and the Constant score-total was 83.78 ± 7.50, also statistically significant improvement (Table 3). Additionally, at 28 days after treatment (V3), VAS-P1 was 2.38 ± 1.96, VAS-P2 was 2.75 ± 1.95, VAS-P3 was 1.81 ± 1.68, UCLA score was 30.13 ± 3.59, SPADI-total was 19.76 ± 11.41, and Constant score-total was 88.05 ± 9.03; the changes of each indicator over time in all evaluation items showed a significant difference and improvement (Table 3, Figure 5).

### 3.3. Comparison of Outcome Measures between the Two Treatment Groups

Regarding the difference (Δ) between the pretreatment (V1) and immediate posttreatment (V2) results in the HIPER-500® group, as a primary outcome, VAS-P1 was measured as 1.39 ± 1.24, VAS-P2 (Δ) was 1.89 ± 1.47, and VAS-P3 (Δ) was 1.06 ± 1.35. As a secondary outcome, the UCLA score (Δ) was measured as −5.39 ± 3.47, SPADI-total (Δ) was 16.98 ± 12.85, and Constant score-total (Δ) was −10.32 ± 11.63. In the SonoStim® group, as a primary outcome, VAS-P1 was measured as 1.69 ± 1.30, VAS-P2 (Δ) was 1.94 ± 1.29, and VAS-P3 (Δ) was 1.25 ± 1.53. As a secondary outcome, UCLA score (Δ) was measured as −3.25 ± 4.37, SPADI-total (Δ) was 14.51 ± 13.68, and Constant score-total (Δ) was −11.69 ± 7.69, with no difference between the two groups (*p* ≥ 0.05) (Table 4, Figure 5).

Regarding the difference (Δ) between the pretreatment (V1) and at 28 days after treatment (V3) in the HIPER-500® group, as a primary outcome, VAS-P1 was measured as 1.72 ± 1.81, VAS-P2 (Δ) was 2.72 ± 1.64, and VAS-P3 (Δ) was 1.28 ± 1.78. As a secondary outcome, UCLA score (Δ) was measured as −4.61 ± 3.82, SPADI-total (Δ) was 19.49 ± 15.33, and Constant score-total (Δ) was −11.37 ± 10.15. In the SonoStim® group, as a primary outcome, VAS-P1 was measured as 2.00 ± 1.83, VAS-P2 (Δ) was 2.38 ± 1.89, and VAS-P3 (Δ) was 1.63 ± 2.25, and as a secondary outcome, UCLA score (Δ) was measured as −3.31 ± 4.25, SPADI-total (Δ) was 16.77 ± 14.60, and Constant score-total (Δ) was −15.96 ± 10.71, with no difference between the two groups (*p* ≥ 0.05) (Table 4, Figure 5).

### 3.4. Adverse Effects

Reported adverse events or voluntary reports of adverse reactions were observed in four patients in the HIPER-500® group and in four patients in the SonoStim® group. None of them was considered a severe adverse event. All four adverse reactions in the HIPER-500® group were redness of the treated area that were treated as the first degree burns without observing further sequelae during follow-up. All four patients participated until the end of the study. Three out of the four adverse events in the SonoStim® group were also redness of the treated area, and the remaining event was an unpleasant tingling sensation.

## 4. Discussion

We investigated how a the newly developed therapeutic deep heating device (HIPER-500®) affects pain relief and function recovery in patients with shoulder pain and compared the outcomes with ultrasound therapy, which is currently used for deep heat treatment. In the HIPER-500® group, the VAS, UCLA, SPADI, and Constant score results all showed improvement, and treatment was still effective after 28 days. In addition, there was no statistically significant difference in the treatment results immediately after treatment and 28 days after treatment for HIPER-500® when compared with the treatment results for ultrasound therapy.

Conservative methods for treating shoulder pain include physical therapies such as hyperthermia, UV therapy, electrotherapy (TENS, ionophoresis, and interferential current), and alternative therapies (vibration, light and laser therapy, and extracorporeal shock wave therapy). High-frequency heat therapy devices have been used as a method for alternative cancer treatment. Recently, their use has been expanded to the musculoskeletal system. High-frequency thermal therapy devices can be divided into CET high-frequency treatment devices and RET high-frequency treatment devices, depending on the mechanism with which they generate heat in the body [13, 25]. In the CET technique, a voltage is applied to the ceramic transducer to generate heat. Since heat is generated only on the one side of the ceramic transducer, there is only a minimal deep diathermic effect, and heat is mostly generated in the tissue located near the epidermis, forcing tissues with a high content of electrolytes to respond, such as muscle and soft tissue. This setting is mostly used for skin care and beauty treatment [13]. Takahashi et al. studied 37 patients with lower back pain due to various causes, using the conventional 2450 MHz ultrashort wave technique and the newly introduced 0.65 ± 0.05 MHz CET therapy, and demonstrated a statistically significant improvement of pain [26]. RET, on the other hand, is more effective in generating deep heat and is effective in tissues with higher resistance such as bone, tendons, and joints and in relieving pain in joint tissues, including ligaments [13, 25]. Thereafter, clinical studies using CRET techniques, CET with Resistive Electric Transfer (RET) techniques, which add effects to bones, tendons, and joints in response to higher resistance tissues, have been performed [13, 27–31].

Unlike conventional ≤1 MHz HIPER-500® machines, CRET was used to combine the advantages of CET and RET at a frequency of 4.4 MHz. This has the advantage of maximizing the effect of heat-induced pain control by improving the permeability of heat and generating strong heat at a high frequency. Additionally, it is possible to regulate the intensity of the energy output and choose either a high, mid, or low level (27, 35, and 45 W/cm^2^). This allows the therapist, in constant interaction with the patients, to control the amount of heat that is applied to the affected area. Finally, the ceramic transducers that touch the skin are insulated to reduce the risk of burns and prevent unpleasant sensations.

The purpose of this study was to investigate whether the newly developed HIPER-500® provides pain relief and functional improvement in patients with shoulder pain. In the HIPER-500® group, both pain and function improved immediately after therapy compared to before treatment. Moreover, one month after the procedure, pain was still significantly reduced and function remained improved. To establish the improvement in shoulder function in detail, the results of the functionality subcategories in the UCLA, SPADI, and Constant scores were compared before with both immediately and one month after treatment, and all showed significant improvement.

The therapeutic efficacy of HIPER-500® was compared with the most widely used ultrasonic therapy device (SonoStim®). There was no significant difference between the HIPER-500® and SonoStim® in the improvement of scores before treatment to immediately after and one month after treatment. When comparing the subjective response to treatment with HIPER-500® and SonoStim®, HIPER-500® patients were more satisfied than those receiving ultrasound therapy because patients could feel a faster temperature increase. Even though not statistically significant, a higher UCLA score was shown with HIPER-500® in terms of patient's satisfaction.

In general, hyperthermia increases chemical activity and the metabolic rate as the temperature in cells and tissue increases, resulting in dilated blood vessels and increased blood flow. This increase in blood flow promotes tissue repair through the introduction of nutrients, oxygen, leukocytes, and antibodies, while the increase in vascular permeability aggregates granulocytes and macrophages into lesions, making it easier to remove toxins and necrotic debris. Moreover, hyperthermia promotes the suppression of chronic inflammatory reactions by inhibiting the activity of several enzymes involved in the inflammatory response (collagenase, oxygenase, and so on), which leads to pain relief and improved function [32, 33]. Furthermore, the sensory nerve conduction velocity of the afferent nerve fiber, which transmits the pain signal, is reduced, and the pain threshold is raised, increasing the pain relief effect by reducing the pain input explained [34]. This analgesic effect of heat is thought to be explained by the gate control theory proposed by Melzack and Wall [35]. The pain relief and functional improvement effects of HIPER-500® are enhanced by the inhibited transmission of pain by the Aß nerves activated by deep heat stimuli delivered to the affected area, thereby increasing the tolerance and threshold of pain. Overall, deep heat may increase the extensibility of deep tissues such as tendons and muscles, resulting in improved physical function of the shoulder [36]. Regarding the stability and side effects of the high-frequency device, four patients in the HIPER-500® group experienced redness of the treatment site. These patients did not have a sense of resistance to the thermal sensation; therefore, the temperature increase was not expressed. The skin redness was detected by the therapist, and thereafter, the intensity was reduced, and the contact time was shortened. Afterwards, the redness disappeared, and the participant completed the study without any further sequelae. There was no other adverse effect. In the subject in the SonoStim® group who experienced left shoulder pains and discomfort, this sensation may have been a result of reversible changes in peripheral nerves caused by ultrasonic devices. This is based on histologic examinations showing a reversible irritation of peripheral nerve fibers when applying therapeutic concentrations of ultrasound to the limbs of Guinea pigs [37]. However, this sensation was not considered as a significant side effect. It should be noted that, in the HIPER-500® group, no neurological symptoms other than 1 degree burns due to transient peripheral nerve stimulation was observed, which was shown in the SonoStim® group. The incidence of side effects was lower in the HIPER-500® group (4/18, 22.2%) than that in the SonoStim® group (4/16, 25%).

The limitations of this study include its small sample size even though the sample size was first calculated and deemed suitable for the comparative study. Second, follow-up was performed for only up to one month after treatment as there was no subsequent long-term follow-up. Finally, the effects of different shoulder diseases on the outcome measures were not analyzed. Although the disease was classified by ultrasound evaluation before treatment, the resulting numbers of comparable samples were too small for meaningful analysis.

## 5. Conclusions

In this study, a high-frequency therapeutic deep heating device (HIPER-500®) led to a reduction in pain and improved function in patients with shoulder pain and dysfunction. There was no difference in the outcome measures when compared with ultrasound as an established deep heat treatment method. HIPER-500® may be a new alternative to deep heat treatment for shoulder disease.

## Figures and Tables

**Table 1:** Two different deep heat treatment modes.

	SonoStim® group (*n*=16)	HIPER-500® group (*n*=18)
Mode	2 W/cm^2^, 0.8 MHz	35 W/cm^2^, 4.4 MHz
Frequency and duration	10 min per session, for a total of ten sessions (five days a week for two weeks)	

**Table 2:** Baseline characteristics of 34 patients in the prospective randomized controlled trial assessing deep heat therapy.

Variables	HIPER-500® (*n*=18)	SonoStim® (*n*=16)	*p* value
Age (yrs)	47.83 ± 12.28	46.75 ± 11.50	0.793
Gender (male : female)	8 : 10	4 : 12	0.236
VAS-P1	4.17 ± 2.38	4.38 ± 0.96	0.736
VAS-P2	6.17 ± 1.86	5.13 ± 1.26	0.068
VAS-P3	2.89 ± 2.59	3.44 ± 1.59	0.469
UCLA score	24.28 ± 3.92	26.81 ± 3.54	0.058
SPADI-total	41.88 ± 22.09	36.53 ± 14.84	0.419
Constant score-total	70.04 ± 16.23	72.09 ± 10.88	0.673

VAS, Visual Analogue Scale; VAS-P1, VAS pain at the current moment; VAS-P2, VAS pain with shoulder movement; VAS-P3, VAS pain in the resting position of the shoulder; UCLA score, University of California at Los Angeles score; SPADI, Shoulder Pain and Disability Index.

**Table 3:** Changes in outcome measures after deep heat treatment in both groups (*n*=34).

	HIPER-500® (*n*=18)				SonoStim® (*n*=16)			
	V1	V2	V3	*p* value	V1	V2	V3	*p* value
VAS-P1	4.17 ± 2.38	2.78 ± 2.13	2.44 ± 2.04	0.012^*∗*^	4.38 ± 0.96	2.69 ± 1.40	2.38 ± 1.96	≤0.001^*∗*^
VAS-P2	6.17 ± 1.86	4.28 ± 2.27	3.44 ± 2.18	0.001^*∗*^	5.13 ± 1.26	3.19 ± 1.72	2.75 ± 1.95	≤0.001^*∗*^
VAS-P3	2.89 ± 2.59	1.83 ± 2.09	1.61 ± 1.72	0.001^*∗*^	3.44 ± 1.59	2.19 ± 1.33	1.81 ± 1.68	0.012^*∗*^
UCLA score	24.28 ± 3.92	29.67 ± 3.31	28.89 ± 3.23	≤0.001^*∗*^	26.81 ± 3.54	30.06 ± 3.19	30.13 ± 3.59	0.013^*∗*^
SPADI-total	41.88 ± 22.09	24.91 ± 17.14	22.39 ± 15.52	0.005^*∗*^	36.53 ± 14.84	22.03 ± 12.65	19.76 ± 11.41	0.001^*∗*^
Constant-total	70.04 ± 16.23	80.36 ± 11.60	81.41 ± 14.50	≤0.001^*∗*^	72.09 ± 10.88	83.78 ± 7.50	88.05 ± 9.03	≤0.001^*∗*^

VAS, Visual Analogue Scale; VAS-P1, VAS pain at the current moment; VAS-P2, VAS pain with shoulder movement; VAS-P3, VAS pain in the resting position of the shoulder; UCLA score, University of California at Los Angeles score; SPADI, Shoulder Pain and Disability Index; V1, visit 1; V2, visit 2; V3, visit 3; ^*∗*^*p* < 0.05.

**Table 4:** Comparison of outcome parameters between the two deep heat treatment groups.

	HIPER-500® (*n*=18)	SonoStim® (*n*=16)	*p* value
V1-V2			
VAS-P1	1.39 ± 1.24	1.69 ± 1.30	0.488
VAS-P2	1.89 ± 1.47	1.94 ± 1.29	0.431
VAS-P3	1.06 ± 1.35	1.25 ± 1.53	0.798
UCLA score	−5.39 ± 3.47	−3.25 ± 4.37	0.175
SPADI-total	16.98 ± 12.85	14.51 ± 13.68	0.448
Constant-total	−10.32 ± 11.63	−11.69 ± 7.69	0.692
V1–V3			
VAS-P1	1.72 ± 1.81	2.00 ± 1.83	0.659
VAS-P2	2.72 ± 1.64	2.38 ± 1.89	0.570
VAS-P3	1.28 ± 1.78	1.63 ± 2.25	0.619
UCLA score	−4.61 ± 3.82	−3.31 ± 4.25	0.355
SPADI-total	19.49 ± 15.33	16.77 ± 14.60	0.600
Constant-total	−11.37 ± 10.15	−15.96 ± 10.71	0.208

VAS, Visual Analogue Scale; VAS-P1, VAS pain at the current moment; VAS-P2, VAS pain with shoulder movement; VAS-P3, VAS pain in the resting position of the shoulder; UCLA score, University of California at Los Angeles score; SPADI, Shoulder Pain and Disability Index; V1, visit 1; V2, visit 2; V3, visit 3.

## Data Availability

The data used to support the findings of this study are available from the corresponding author upon request.
